# The Case of Governor Sullivan of Massachusetts

**Published:** 1810-04

**Authors:** J. C. Warren


### Medical and Physical Journal.

VOL. XXIII.] APRIL, 1810. [NO. 134.

*Printed for R. PHILLIPS, by W. Thorne, Red Lion Court, Fleet Street, London.*

### *The* CASE *of Governor* SULLIVAN *of Massachusetts.*

[ Communicated by Dr. J. C. WARREN. ]

THE symptoms of organic disease of the heart, are marked with extraordinary clearness in the following case. The opportunity for observing them was very favourable; and there was every incitement to close observation, which could arise from the important and interesting character of the patient. These advantages will justify an uncommon minuteness in the detail of the case; especially, as the most accurate knowledge of a complaint is obtained from a successive view of its stages.

The late Governor of this commonwealth was endowed with most vigorous powers of mind and body. At the age of 16, he was attacked with fits of epilepsy, which first arose from a sudden fright, received on awaking from sleep in a field, and beholding a large snake erecting its head over him. As he advanced in life, they became more frequent, and were excited by derangement of the functions of the stomach, often by affections of the mind, by dreams, and even by the sight of the reptile which first produced the convulsions.

At the commencement of the American revolution, he became deeply engaged in public affairs; and from that time devoted himself to intense application to business, with which the preservation of his health was never allowed to interfere. In the expedition against Rhode Island, an attack of inflammation of the lungs had nearly proved fatal to him.

In the beginning of the year 1807, he suffered severely from the epidemic catarrh; and a remarkable irregularity of the pulse was then perceived to be permanent, though there is some reason to believe, that this irregularity had previously existed, during the fits of epilepsy, and for a few days after them. In the summer, while he was apparently in good health, the circulation in the right arm was suddenly and totally suspended; yet, without loss of motion or sensation. This affection lasted from noon till midnight, when it as suddenly ceased, and the circulation was restored. In the autumn he was again seized with the influenza, which continued about three weeks, leaving a troublesome cough of two or three months' duration, and a slight occasional difficulty of breathing, which at that time was not thought worth attention. Soon after, in November, he had one or two singular attacks of catarrhal affection of the mucous membrane of the lungs, which commenced with a sense of suffocation, succeeded by cough and an expectoration of cream coloured mucus, to the quantity of a quart in an hour, with coldness of the extremities, lividity of the countenance, and a death-like moisture over the whole body. These attacks lasted six or eight hours, were relieved by emetics, and disappeared, without leaving a trace behind.

At this time, he began to complain of palpitations of the heart; yet, it is probable, that he had been affected with these before, since he was unaccustomed to mention any complaint, which was not sufficiently distressing to require relief. He experienced a difficulty of respiring, as he ascended the stairs, and became remarkably susceptible of colds, from slight changes of clothing, moisture of the feet, or a current of cold air. His sleep was unquiet in the night, aud attended with very profuse perspiration; and, in the latter part of the day, a troublesome heaviness occurred. The sanguiferous vessels underwent an extraordinary increase, or, at least, became remarkably evident. The pulsation of the carotid arteries was uncommonly strong; the radial arteries seemed ready to burst from their sheaths; the veins, especially the jugulars, in which there was often a pulsatory motion, were every where turgid with blood. The countenance was high coloured, and commonly exhibited the appearance of great health; but, when he was indisposed from catarrh, this florid red changed to a livid colour; which also, after an attack of epilepsy, was observable for two or three days on the face and hands. This livid hue was often attended, under the latter circumstances, with something like ecchymosis over the face, at first formidable in its aspect, and gradually subsiding, till it had the general appearance of an eruption, which also soon vanished.

These symptoms increased, almost imperceptibly, during the five first months of the year 1808. Much of this time was passed in close application to official duties; and it seemed seemed that a constant and regular occupation of the mind had the effect of obviating the occurrence of any paroxysm of disease, as well of epilepsy, as of difficult respiration; and that a very sudden and disagreeable impression generally produced either one or the other. There were, indeed, independently of such circumstances, some occasional aggravations of those symptoms. Some nights, for example, were passed in sitting up in bed, under a fit of asthma, as it was called; sometimes the mind became uncommonly impatient and irritable; the body gradually emaciated; yet the appetite and digestive functions remained principally unimpaired; and persons around were not sensible of any material alteration in the condition of the patient.

On the approach of warm weather, in June, the violence of the symptoms increased. Paroxysms of dyspnœa occurred more frequently, and were more distressing. They commenced with symptoms of slight febrile affection, such as hot skin, hard, frequent, and more irregular pulse, disordered tongue, loss of appetite, and derangement of the digestive functions. This kind of paroxysm lasted two or three days. Evacuations of blood from the nose and hæmorrhoidal vessels, which before rarely occurred, became frequent; a fulness at the upper and right side of the abdomen was sometimes perceptible, formed apparently by temporary enlargement of the liver; the difficulty in ascending an eminence increased sensibly. In the intervals of these attacks, which were variable, but generally continuing ten or twelve days, the strength was frequently good, and accompanied by a great flow of spirits, and an aptitude, or rather ardour, for business.

Such was the course of this complaint until the latter part of August, when a very severe paroxysm occurred. It commenced, like the former, with febrile symptoms, but those more violent than before. The countenance became high coloured; the dyspnœa excessive, and rendered almost suffocating by a slight movement, or attempt to speak; the pulse hard, very irregular, intermittent, and vibrating; and the digestive functions were suspended. These symptoms soon increased to the highest degree. The respiration was so distressing, as to produce a wish for speedy death; the eyes became wild and staring. No sleep could be obtained; for, after dosing a short time, he started up in violent agitation, with the idea of having suffered a convulsion. During the few moments of forgetfulness, the respiration was sometimes quick and irregular, sometimes slow, and frequently suspended for the space of twenty-five, and even so long as fifty seconds. At the end of three days, the febrile heat was less permanent; the red colour of the face changed to a death-like purple; the hands and face were cold, and covered with an adhesive moisture; the hardness of the pulse diminished, and a degree of insensibility took place. I seized this opportunity to examine the region of the heart, which had not been done before, from fear of alarming the active and irritable mind of the patient. The heart was perceived palpitating, obscurely, about the 7th and 8th ribs; its movements were very irregular, and consisted in one full stroke, followed by two or three indistinct strokes, and sometimes by an intermission, corresponding with the pulse at each wrist. The pulsation was felt more distinctly in the epigastric region. During this paroxysm, a recumbent posture was very uneasy, and the patient uniformly preferred sitting in a chair. When the recumbent posture was assumed, the head was much raised, inclined to the right side, and supported by the hand; the knees were drawn up as much as possible. He could not bear an horizontal posture; nor did he ever lie on the left side, except a short time after the application of a blister. At the end of the fifth day, his sufferings abated, but the sudden affusion of a small portion of a cold liquid on the head produced a severe fit of epilepsy. This was followed by a return of the symptoms equally distressing, and more durable, than in the first attack.*

This violent agitation gradually subsided, and was followed by a pleasant calm. The natural functions resumed their ordinary course; his appetite returned; his enjoyment of social intercourse was unusually great; and he amused and instructed his friends by the immense treasures of information, which his talents and observations had afforded him, and which, he seemed to feel, would soon be lost. At the end of September, the feet began to swell, and after some time, the enlargement extended up to the legs and

* During this time, it was thought adviseable to acquaint his friends, that an organic disease of the heart existed, which doubtless consisted in an ossification of the semilunar valves of the aorta, attended, perhaps, by enlargement of the heart; that the disease was beyond the reach of art, and would prove fatal within three months, possibly very soon; that if it lasted so long, it would be attended by frequent recurrences of those distressing symptoms, general dropsical affections, and an impaired state of the mental faculties.

and thighs, and increased to an extraordinary degree; the abdomen next swelled, and, after it, the face. Toward the end of October, there were some indications of water in the chest; there was a constant shortness and difficulty of breathing; the cough, till now rare, became more frequent and troublesome; the contraction of the thoracic cavity rendered the action of the heart more painful, to that beside an uniform stricture across the breast, he sometimes described a dreadful sensation like twisting of the organs in the thorax. He suspected the existence of water there, and was inclined to consider it as his primary disease, but was easily convinced of the contrary. At one time he had a suspicion of a complaint of the heart, and, although he had never heard of a disease of that organ, slightly intimated it to one of his friends, and mentioned a sensation he had experienced in the chest, which he compared to a fluid driven through an orifice too narrow for it to pass freely. In this month, beside the dropsical affections and increase of cough, he had occasional painful enlargements of the liver, frequent starting up from sleep, a slight degree of dizziness, a great disposition for reveries, and sometimes extraordinary illusions, one of which was, that he was two individuals, each of whom was dying of a different disease. This idea often occurred, and gave him much uneasiness. He was also afflicted with long continued frightful dreams, and sometimes a slight delirium.

After the use of much medicine, on the 6th of November, the effused fluids began to be absorbed, and passed out through the urinary organs with such rapidity, that on the 12th the dropsical enlargements had nearly disappeared. The pulse was much reduced, in hardness and frequency, by the medicine, and, as it fell, he became more easy. On the 10th, the state legislature convened, and the call of business roused, like magic, the vigor of his mind; and the symptoms of his disease almost disappeared. During this session, he made little complaint, dictated many important communications, and attended to all the duties of his office, without neglecting the most minute. As soon as the legislature adjourned, he declared, that his work was finished, and that he had no desire to remain longer in this world. He entreated that no farther means should be used to prolong his existence, and immediately yielded himself to the grasp of disease, which appeared waiting with impatience to inflict its agonies.

From this moment, the distressing difficulty of breathing had very slight remissions. The consequent disposition to incline the superior part of the body forward, for the purpose of facilitating respiration, increased so much, that he frequently slept with his head reposed on his knees. The cough became occasionally very violent, and was always attended with an expectoration of a brown coloured mucus, sometimes tinged with blood. The abdominal viscera lost their activity. The face was sometimes turgid and high coloured, at other times, pallid and contracted. A gradual abolition of the powers of the mind ensued, with a low delirium, and two short fits of phrenzy. The state of the circulation was very variable; the pulse at the wrists principally hard and vibrating, rarely soft and compressible; the less pulsations becoming more indistinct, and, at length, scarcely perceptible. No perfectly distinct beat of the heart was felt, but a quick undulating motion, not corresponding with the pulse at the wrist. Three days before death, the arteries assumed this undulatory motion, corresponded with the motion of the heart, and, for forty-eight hours, lost the irregularity of pulsation.*

Once or twice the expiring faculties brightened. On the 30th of November he awoke, as if from death, conversed very pleasantly for two or three hours, and humorously described scenes, which he had witnessed in his youth.

On the 4th of December, came on the second attack of furious delirium. Insensibility, and great prostration of strength, ensued. The respiration became very slow, and obstructed by the accumulation of mucus in the lungs; the pulse very intermittent, then regular, and finally fluctuating. A hiccough commenced; coldness of the extremities and lividity of the face followed, and continued three days before death. On the 9th, the incurvated posture was relinquished, and the head sunk back upon the pillow; the respirations

---

* The celebrated Morgagni has recorded some cases of organic disease of the heart discovered by dissection, the symptoms of which do not exactly accord with those observed in this and the succeeding cases. It should be remembered, however, that many of the subjects of those cases were not examined by him, while living, and others but a very short time before death. But it appears, that, in the last stage of this disorder, some of the most important symptoms may be materially changed, especially the state of the pulse, dyspnœa and palpitations. Thus in the case related above, and in some others, the pulse became regular, the palpitations subsided, and the dyspnœa was less observable. The cases of that accurate anatomist, therefore, are not so contradictory of those related here, as might at first be imagined.

respirations then diminished in frequency, till they became only two in a minute; and at the end of twenty-four hours they very gradually ceased.*

Dissection, nine hours after death. *External Appearance.*—The whole body was much emaciated; the face pale and contracted. The hands were slightly œdematous. Discolourations, answering to the ribs, were observed on the thorax; many small purple spots, hard and prominent on the back; excoriations on the nates; and purple spots, resembling incipient mortification on the heel and toe.

Thorax.—The integuments of the thorax were free from fat: the cartilages of the ribs ossified iu various degrees, some perfectly, others slightly. Upon laying open the cavity of the thorax, it was found to contain about three pints of water, the proportion being greatest on the left side.

The lungs were contracted into a smaller compass than usual, and were very firm to the touch. Their colour anteriorly was whitish, with small distinct purple spots; posteriorly, of a deep red, with similar spots. The right lobe adhered closely to the pericardium; it also adhered to the pleura costalis, by a great number of strong cords, which seemed to be elongations of the original adhesions. Some of them were nearly as hard as ligament, and many an inch in length. Internally, the lungs presented a very compact structure. Their cells were crowded with mucus, and their vessels filled with black blood, partly fluid, and partly coagulated. Some portions were firmer and more condensed than others, but no tubercles were discovered.

The pericardium, viewed externally, appeared very large, and occupied almost the whole space behind the opening, formed by removing the sternum and cartilages of the ribs. It was situated principally on the left side, and contained about double the usual quantity of water; but was principally filled by the enlarged heart, to which it adhered anteriorly about two inches, near its base. Its parietes were, in every part, very much thickened and hardened.

The heart presented nearly its usual colour and form, excepting on its anterior surface, which was somewhat discoloured by coagulated lymph. It was enlarged in bulk, to

* Governor Sullivan was born December 4th, 1744, and died December 10th, 1808.

to at least one half more than the healthy size. The auricles and ventricles contained coagulated blood. The tricuspid valves were in a sound state. The left auricle was double the usual size. The left ventricle was enlarged, about three times thicker and much firmer than usual. The mitral valves were very much thickened, and near the insertion of their columnæ, which were sound, cartilaginous, so that they were quite rigid, and the opening made by them, from the auricle to the ventricle, was scarcely large enough to admit the passage of a finger. The semilunar valves of the aorta were ossified at their bases and apices, and the portion intermediate, between the base and apex, partly ossified, and partly cartilaginous, so as to render the valves very rigid. The aorta was at least one half larger than usual, especially at its arch. The arteria innominata, the carotid, and subclavian arteries, were uncommonly large and thick. The coronary arteries were considerably ossified.

Abdomen.—The omentum was destitute of fat. The stomach distended with flatus on the pyloric side; its cardiac extremity, lying under the liver, was pressed down and contracted. The liver was shrunk; its tunic corrugated, as if it had been distended, and bearing marks of inflammation; its substance harder than usual; its vessels, when divided, pouring out liquid black blood. The gall-bladder was filled with bile. The kidneys were thicker, and more irregular in form, than is common. The abdominal cavity contained some water.

Head.—The bones of the cranium were unusually thick. The dura mater, which was thickened, and in many places bore marks of former inflammation, adhered to the bone at the vertex. On its internal surface, near the longitudinal sinus, there was a small ossified portion, half an inch long, and the eighth of an inch thick. The convolutions of the brain were narrow, and very strongly marked. The pia mater bore marks of pretty extensive inflammation, and adhered to the dura mater at the vertex. The cortical substance ran deep into the medullary part of the brain. The ventricles contained about double the usual quantity of water; their parts were all remarkably well defined. The vessels of the pia mater, over the corpora striata, were unusually injected with blood. The velum interpositnm was very firm; the plexus choroides uncommonly thick, but pale; the opening from the right to the left ventricle large. The vessels of the brain were generally not much filled with blood.

The

The blood appeared every where fluid, except in some portions of the lungs, and in the cavities of the heart. It was very dark coloured, perhaps more than ordinarily thin, and oozed from every part which was cut.

The cellular membrane, in all dependent parts, effused, when cut, a serous fluid.

---

*This industrious and accurate Practitioner has just published, at Boston, a Collection of Cases of Organic Diseases of the Heart, from a comparison of which with other Diseases in some respects resembling them, he deduces the following Remarks.*

Morbid changes in the organization of the heart are so frequent, as to have attracted the observation of those who have devoted any attention to the study of morbid anatomy. Derangements of the primary organ of the circulation cannot exist without producing so great disorder of the functions of that and of other parts, as to be sufficiently conspicuous by external signs; but, as these somewhat resemble the symptoms of different complaints, especially of asthma, phthisis pulmonalis, and water in the thorax, it has happened, that each of these has been sometimes confounded with the former. The object of the following statement of cases is to shew, that, whatever resemblance there may be in the symptoms of the first, when taken separately, to those of the latter diseases, the mode of connection and degree of those symptoms at least is quite dissimilar; and that there are also symptoms peculiar to organic diseases of the heart, sufficiently characteristic to distinguish them from other complaints.

The first notice of disorder is commonly from an irregular and tumultuous movement of the heart, which occurs some time before any perceptible derangement of the other functions. This irregularity slowly increases, and arrives at its height before the strength of the patient is much impaired, at least in the cases which I have noticed; and as the vigour of the patient lessens, the force of the palpitations diminishes. These palpitations are often so strong, as to be perceptible to the eye at a considerable distance. They are seldom most distinct in the place where the pulsation of the heart is usually felt. Sometimes they are perceived a little below; often in the episgastric region; and not unfrequently beneath, and on the right side of the sternum.

After the palpitations have lasted some time, a little difficulty of breathing, accompanied with sighing, is perceived, especially on any great exertion, ascending an eminence, eminence, or taking cold, of which there is an uncommon susceptibility. This dyspnœa becomes, as it increases, a most distressing symptom. It is induced by the slightest cause; as by an irregularity in diet, emotions of the mind, and especially movement of the body; so that on ascending stairs quickly, the patient is threatened with immediate suffocation. It occurs at no stated periods, but is never long absent, nor abates much in violence during the course of the disease. It is attended with a sensation of universal distress, which perhaps may arise from the circulation of unoxygenated blood, or the accumulation of carbon in the system; for the countenance becomes livid, and the skin, especially that of the extremeties, receives a permanent dark colour. This dyspnœa soon causes distress in lying in an horizontal posture. The patient raises his head in bed, gradually adding one pillow after another, till he can rarely, in some cases never, lie down without danger of suffocation; he inclines his head and breast forward, and supports himself upon an attendant, or a table placed before him. A few hours before death the muscular power is no longer capable of maintaining him in that posture, and he sinks backward. The dyspnœa is attended with cough, sometimes through the whole of the disease, sometimes only at intervals. The cough varies in frequency. It is always strong, and commonly attended with copious expectoration of thick mucus, which, as the disease advances, becomes brown coloured, and often tinged with blood; a short time before death it frequently consists entirely of black blood.

The changes in the phenomena of the circulation are very remarkable, The sanguiferous system is increased in capacity; the veins, especially, are swelled with blood; the countenance is high coloured, except in fits of dyspnœa, when it becomes livid; and it is very frequently puffed, or turgid. The brightness of the eyes, dizziness, which is a common, and head-ache, which is a frequent symptom, and in some cases very distressing, are probably connected with these changes. The motions of the heart, as has already been stated, are inordinate, irregular, and tumultuous. The pulse presents many peculiarities. In some cases, probably where there is no obstruction in the orifices of the heart, it remains tolerably regular, and is either hard, full, quick, vibrating and variable, or soft, slow, compressible and variable. Most commonly, perhaps always, when the orifices of the heart are obstructed, it it is vibrating, very irregular, very intermittent, sometimes contracted and almost imperceptible, very variable, often disagreeing with the pulsation of the heart, and sometimes differing in one of the wrists from the other.

The functions of the brain suffer much disturbance. Melancholy, and a disposition for reverie, attend the early stages of the complaint; and there is sometimes an uncommon irritability of mind. The dreams become frightful, and are interrupted by sudden starting up in terror. Strange illusions present themselves. The mental faculties are impaired. The termination of the disease is attended with slight delirium; sometimes with frenzy, and with hemiplegia.

The abdominal viscera are locally, as well as generally affected. Although the digestive functions are occasionally deranged, the appetite is at some periods remarkably keen. The action of the intestines is sometimes regular, but a state of costiveness is common. The liver is often enlarged, probably from accumulation of blood. This distention is attended with pain, varies much, and, in all the cases I have seen, has subsided before death, leaving the coats of the liver wrinkled, flaccid, and marked with appearances of inflammation, caused by the distention and pressure against the surrounding parts. An effect of the accumulation of blood in the liver, and consequently in the mesenteric veins, is the frequent discharge of blood from the hæmorrhoidal vessels. This occurs both in the early and late stages of the disease, and may become a formidable symptom. Evacuations of blood from the nose are not uncommon.

Dropsical swellings in various parts of the body succeed the symptoms already enumerated. They commence in the cellular membrane of the feet, and gradually extend up the legs and thighs; thence to the abdominal cavity, to the thorax, sometimes to the pericardium, to the face and superior extremities; and, lastly, to the ventricles and meninges of the brain. These collections of water may be reabsorbed by the aid of medicine; but they always return, and attend, in some degree, the patient's death.

There is no circumstance more remarkable in the course of this complaint, than the alternations of ease and distress. At one time the patient suffers the severest agonies, assumes the most ghastly appearance, and is apparently on the verge of death; in a day or a week after, his pain leaves him, his appetite and cheerfulness return, a degree of vigour is restored, and his friends forget that he has been ill.

ill. The paroxysms occasionally recur, and become more frequent, as the disease progresses. Afterwards the intermissions are shorter, and a close succession of paroxysms begins. If the progress of the complaint has been slow, and regular, the patient sinks into a state of torpor, and dies without suffering great distress. If, on the contrary, its progress has been rapid, the dyspnœa becomes excessive; the pain and stricture about the præcordia are insupportable; a furious delirium sometimes succeeds; and the patient expires in terrible agony.

Such are the symptoms which a limited experience has enabled me to witness. Others, equally characteristic of the disease, may probably exist.

From this description of the symptoms it would appear, that there could be no great difficulty in distinguishing this from other diseases; yet probably it has sometimes been confounded with asthma, and very frequently with hydrothorax. Some may think, that there is no essential difference in the symptoms of these diseases. The resemblance between them, however, is merely nominal.

The cough in hydrothorax, unlike that which attends organic diseases of the heart, is short and dry; the dyspnœa constant, and not subject to violent aggravations. An uneasiness in an horizontal posture attends it, but no disposition to incurvate the body forward. These are some of the points, in which these two diseases slightly resemble each other. Those, in which they totally differ, are still more numerous; but as most of them have been already mentioned, it is unnecessary to indicate them here.

It is probable, that the two diseases commonly arise in patients of opposite physical constitutions; the hydrothorax in subjects of a weak relaxed fibre; the organic diseases of the heart in a rigid and robust habit. The subjects of the latter affection, in the cases which have fallen under my observation, were, with the exception of one or two instances, persons of ample frame, and vigorous muscularity, and who had previously enjoyed good health. In nearly all these cases the collection of water was principally on one side, yet the patients could lie as easily on the side where there was least fluid, as on the other; which in the opinion of most authors, is not the case in primary hydrothorax. It should also be observed, that, in many of the cases, there was only a small quantity of water in the chest, and that in neither of them was there probably sufficient to produce death. May not primary hydrothorax be much less frequent than has commonly been imagined?

Idiopathic

Idiopathic dropsy of the pericardium may, perhaps, produce some symptoms similar to those of organic disease of the heart; but it appears to be an uncommon disorder, and I have had no opportunity of observing it. In the fourth case, a remarkable disposition to syncope, on movement, distinguished the latter periods of the disease, and might have arisen from the great collection of water in the pericardial sac.

The causes of this disease may, probably, be whatever violently increases the actions of the heart. Such causes are very numerous; and it is therefore not surprising, that organic diseases of the heart should be quite frequent. Violent and long continued exercise, great anxiety and agitation of mind,* excessive debauch, and the habitual use of highly stimulating liquors are among them.

The treatment of this complaint is a proper object for investigation. Some of its species, it is to be feared, must for ever remain beyond the reach of art; for it is difficult to conceive of any natural agent sufficiently powerful to produce absorption of the thickened parietes of the heart, and at the same time diminish its cavities; but we may indulge better hopes of the possibility of absorbing the osseous matter and fleshy substance deposited in the valves of the heart and coats of the aorta. A careful attention to the symptoms will enable us to distinguish the disease, in its early stages, in which we may undoubtedly combat it with frequent success.

Although it may not admit of cure, the painful symptoms attending it may be very much palliated; and as they are so severely distressing, we ought to resort to every probable means of alleviating them. Remedies, which lessen the action of the heart, seem to be most commonly indicated. Blood-letting affords more speedy and compleat relief, than any other remedy. Its effect is quite temporary, but there can be no objection to repeating it. The digitalis purpurea seems to be a medicine well adapted to the alleviation of the symptoms, not only by diminishing the impetus of the heart, but by lessening the quantity of circulating fluids. Its use is important in removing

* It has been remarked by the French physicians and particularly by M. Corvisart, physician to the emperor of France, that these organic diseases were very prevalent after the revolution, and that the origin of many cases was distinctly traced to the distressing events of that period.

moving the dropsical collections; and for this purpose it may often be conjoined with mercurials. Expectoration is probably promoted by the scilla maritima, which, in a few cases, seemed also to alleviate the cough and dyspnœa. Blisters often diminish the severe pain in the region of the heart, and the uneasiness about the liver. It has been seen, that the excessive action of the heart sometimes produces inflammation of the pleura and pericardium, and that the distention of the coat of the liver has the same effect upon that membrane in a slighter degree. Vesication may probably lessen those inflammations. When the stomach and bowels are overloaded, a singular alleviation of the symptoms may be produced by cathartics, and even when that is not the case, the frequent use of moderate purgative medicines is advantageous. Full doses of opium are, at times, necessary through the course of the complaint. The antiphlogistic regimen should be carefully observed. The food should be simple, and taken in small quantities, stimulating liquors cautiously avoided, and the repose of body and mind preserved, as much as possible.

